# Single-cell transcriptional profiling of hearts during cardiac hypertrophy reveals the role of MAMs in cardiomyocyte subtype switching

**DOI:** 10.1038/s41598-023-35464-2

**Published:** 2023-05-23

**Authors:** Yi Luan, Guangyu Guo, Ying Luan, Yang Yang, Ruixia Yuan

**Affiliations:** 1 Clinical Systems Biology Research Laboratories, Translational Medicine Center, The First Affiliated Hospital of Zhengzhou University, Zhengzhou, 450052 People’s Republic of China; 2 Department of Neurology, The First Affiliated Hospital of Zhengzhou University, Zhengzhou University, Zhengzhou, 450052 People’s Republic of China; 3 Department of Physiology and Neurobiology, School of Basic Medical Sciences, Zhengzhou University, Zhengzhou, 450001 People’s Republic of China; 4 Clinical Big Data Center, the First Affiliated Hospital of Zhengzhou University, Zhengzhou, 450052 People’s Republic of China

**Keywords:** Cardiovascular diseases, Heart failure

## Abstract

Pathological cardiac hypertrophy is the main predecessor of heart failure. Its pathology is sophisticated, and its progression is associated with multiple cellular processes. To explore new therapeutic approaches, more precise examination of cardiomyocyte subtypes and involved biological processes is required in response to hypertrophic stimuli. Mitochondria and the endoplasmic reticulum (ER) are two crucial organelles associated with the progression of cardiac hypertrophy and are connected through junctions known as mitochondria-associated endoplasmic reticulum membranes (MAMs). Although MAM genes are altered in cardiac hypertrophy, the importance of MAMs in cardiac hypertrophy and the expression pattern of MAMs in certain cardiac cell types require a comprehensive analysis. In this study, we analyzed the temporal expression of MAM proteins in the process of cardiac hypertrophy and observed that MAM-related proteins preferentially accumulated in cardiomyocytes at the initial stage of cardiac hypertrophy and underwent a gradual decline, which was synchronized with the proportion of two cardiomyocyte subtypes (CM2 and CM3). Meanwhile, these subtypes went through a functional switch during cardiac hypertrophy. Trajectory analysis suggested that there was a differentiation trajectory of cardiomyocyte subtypes from high to low MAM protein expression. Distinct regulon modules across different cardiomyocyte cell types were revealed by transcriptional regulatory network analysis. Furthermore, scWGCNA revealed that MAM-related genes were clustered into a module that correlated with diabetic cardiomyopathy. Altogether, we identified cardiomyocyte subtype transformation and the potential critical transcription factors involved, which may serve as therapeutic targets in combating cardiac hypertrophy.

## Introduction

As the first organ formed during embryo development stages, the heart undertakes billions of beats to sustain a well-circulated body with lovely oxygenated blood^1^. Cardiomyocytes undergo hypertrophic growth in response to hypertension, valvular heart disease, and so on and ultimately develop heart failure^2^. Although previous studies implicated a variety of signaling pathways that are closely related to pathological hypertrophy, effective therapy is still insufficient. This situation might be due to several drawbacks. First, pathological hypertrophy involves a complex process, including healthy to hypertrophy with normal ejection fraction (EF), then to hypertrophy with reduced EF, and to a failed state^3^. Second, the heart is composed of several cell types with distinct biological functions^4^. A thorough profile of each cell type function during cardiac hypertrophy is still confusing. A recent study revealed that ACKR1^+^ endothelial cells (ECs) serve as an important cell type in the maintenance of cardiac function via single-cell analysis, emphasizing the importance of revisiting cardiac diseases at single-cell resolution. The pathogenesis of cardiac hypertrophy is sophisticated, and its progression is associated with multiple cellular processes^2,5,6^. In addition, the mechanisms of cardiac hypertrophy remain elusive.

Mitochondrial dysfunction has been implicated in the development and pathogenesis of cardiac hypertrophy^7^. Moreover, previous studies have associated hypertrophic processes with many mitochondrial processes^8^. Aside from the mitochondria, endoplasmic reticulum (ER) dysfunctions have also been implicated in the development of cardiac hypertrophy^9^. The mitochondria and ER have been shown to closely interact with each other, which are designated mitochondria-associated membranes (MAMs) or mitochondria-ER contact sites (MERCs)^10^. To date, the number of proteins that localize MAMs has reached ~ 1000 in the brain and liver^11^. The components in MAM proteins are highly conserved among species and tissues^12^. The functions of MAMs are quite diverse, ranging from calcium transfer, lipid synthesis, and autophagy to oxidative stress. In addition, MAMs are also responsible for inflammasome formation, ER stress and mitochondrial dynamics^12^. MAM-localized proteins play a critical role either in the physical interaction of MAMs or in modulating tethering complexes in MAMs^13^. Tethering complexes include Ca^2+^ channels and apoptotic proteins and act as scaffolds bridging the ER and the mitochondrial membrane^13^. The role of MAMs in the development of cardiovascular diseases has been identified^11,14^. In the course of cardiac hypertrophy, the contact between the ER and mitochondria is changed. When stimulated with a hypertrophic factor, norepinephrine, the distance between the ER and mitochondria is enhanced, which reduces calcium reuptake in cardiac mitochondria and further deteriorates cardiac hypertrophy^15^. Moreover, defects in ER-mitochondria communication and calcium transfer may serve as a precondition for pathological cardiac hypertrophy in aged mice. For instance, cardiac-specific depletion of RYR2 impairs ER-mitochondria calcium exchange and results in spontaneous myocardial hypertrophy as well as fibrous hyperplasia in mice^16^. However, the importance of MAMs in cardiac hypertrophy and the expression pattern of MAMs in certain cardiac cell types still require a comprehensive analysis.

In this study, we analyzed the temporal and spatial expression of MAM proteins in the process of cardiac hypertrophy and assessed the cardiomyocyte subtypes in mediating the process of cardiac hypertrophy. Moreover, we also delineated critical transcription factors involved in cardiac hypertrophy.

## Materials and methods

### Single-cell RNA sequencing (scRNA‑seq) analysis

Single-cell RNA sequence data from heart tissues of transverse aortic constriction (TAC)-model mice were obtained from the Gene Expression Omnibus (GEO) database (GSE120064). Cells expressing < 200 or > 10,000 genes were filtered out for exclusion of noncell or cell aggregates. The data were log‐normalized, highly variable genes were selected using the FindVariableFeatures function, and downstream procedures were performed using the ScaleData and runPCA function. Then, the FindNeighbors and FindClusters functions were used to cluster cells, and uniform manifold approximation and projection (UMAP) with the R package Seurat was used to visualize clusters, as described in the vignettes (https://satijalab.org/seurat/vigne ttes.html). Clusters were annotated by known cell markers.

### MAM score

To score the cells for the MAM gene set, the AddModuleScore function of Seurat was used. The MAM gene set was obtained from the literature^17^ and contained Mfn1, Mfn2, Bcap31, Pacs2, Tespa1, Hspa9, Itpr3, Sigmar1, Vapb, Fis1, Vdac, Mcu, and Ptpip51.

### Enrichment analysis

For pathway enrichment analysis, all cluster-specific markers or differentially expressed genes resulting from the “FindMarkers” function in Seurat were used for gene set variation analysis (GSVA) or gene set enrichment analysis (GSEA) in RStudio^18^. The R package clusterProfiler was used in this analysis with a cutoff q-value of 0.05. The results were generated with ggplot2 as bar plots or GSEA plots, and the volcano plots used to compare the expression levels of differential genes between groups were generated using ggplot2 in R.

### Pseudotime analysis

We used Monocle2 (v2.6.0)13 to study the pseudotime trajectory of cells. The UMI matrix was used as input, and variable genes detected by Seurat were used to build traces.

We used the following criteria to select CM subclusters as input of Monocle: (1) The subclusters should account for > 10% of total CM cells at any time point. (2) There were at least 10 cells supporting the potential conversion between two subclusters. (3) The conversion direction should not conflict with the proportion change of the clusters. Potential cell conversion was calculated according to a previously reported method14. Briefly, a cell of the current stage was removed for Spearman correlation to all the cells in the previous stage. The top 5 correlated cells were considered “potential ancestors”. Then, the cell clusters of these 5 cells were combined and supported by voting. Finally, all ancestor origins of a “current” CM subcluster were calculated based on this approach.

### Weighted gene coexpression network analysis (WGCNA)

We applied WGCNA to our scRNA-seq dataset using the R package scWGCNA according to the online protocol (https://smorabit.github.io/tutorials/9_scWGCNA_tutorial/). Modules were clustered under the following specific module-cutting parameters: module size = 50 genes, deepSplit Score = 4, threshold of correlation = 0.2. Modules with a correlation of > 0.8 were merged.

### Transcriptional regulon analysis

The python version of SCENIC (pySCENIC version 0.10.3) was employed to analyze the regulatory network according to the workflow (https://pypi.org/project/pyscenic/0.6.6/#tutorial), and we collocted a mouse TF gene list and mm9-tss-centered-10 kb-10species database from the resources of pySCENIC (https://github.com/aertslab/pySCENIC/tree/master/resources).

### RNA extraction and real‑time qPCR analysis

The total RNA from heart tissues was extracted with TriZol reagent (Invitrogen, USA), and then reverse-transcribed into cDNA using PrimeScript RT Master Mix (Takara, Japan). SYBR Premix Ex TaqII (Takara, Japan) kit was used for RT-PCR detection on ABI PRISM 7500 Detection System (ABI, USA). The relative expression of genes was normalized by GAPDH. The primers are listed as below: Mfn1; 5′-GTGAGCTTCACCAGTGCAAA-3′, 5′-CACAGTCGAGCAAAAGTAGTGG-3’, Mfn2; 5′- CGAGGCTCTGGATTCACTTC -3′, 5′- CAACCAGCCAGCTTTATTCC-3’, Hspa9; 5′- GTTGGTATGCCAGCAAAACGGC-3′, 5′- CAAGCATCACCATTGGAGGCAC-3’, Fis1; 5′- GATCATCCTCGGATGTAGGG-3′, 5′-GACTGAAATTTCCTTTCAAAATTCC-3’, Actin; 5′- CATTGCTGACAGGATGCAGAAGG-3′, 5′- TGCTGGAAGGTGGACAGTGAGG-3’.

## Results

### MAM-related genes are preferentially expressed in cardiomyocytes of cardiac hypertrophy samples

**Figure 1:**
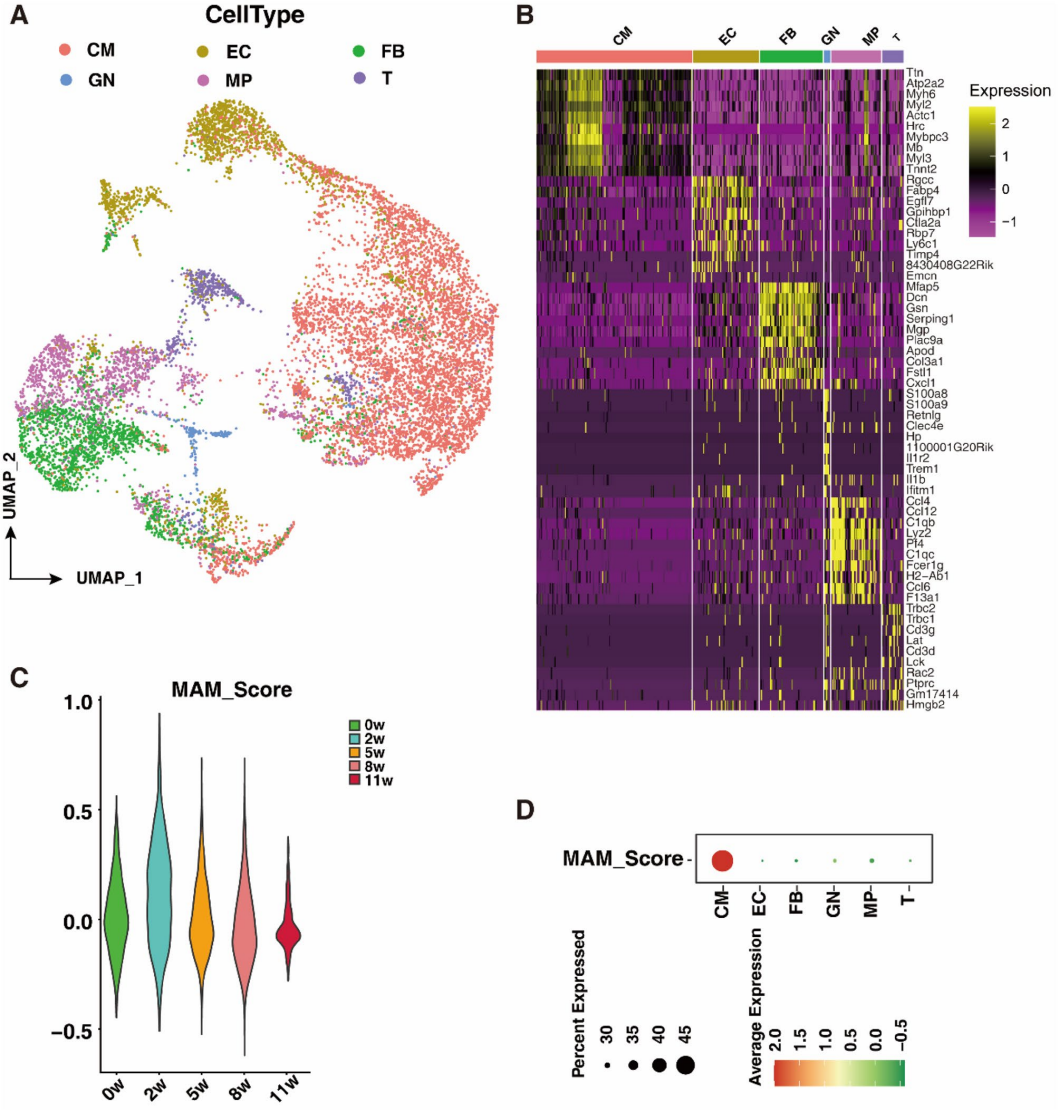
MAM-related genes are preferentially expressed in cardiomyocytes of cardiac hypertrophy samples. (**A**) Uniform Manifold Approximation and Projection (UMAP) showing cell types isolated from different stages of cardiac hypertrophy according to the expression of specific markers. (**B**) Heatmap showing differentially expressed genes (DEGs) in each cell type. (**C**) MAM scores in heart tissues at different stages of cardiac hypertrophy. (**D**) Average MAM gene expression in CMs, ECs, FBs, GNs, MPs and T cells of cardiac hypertrophy. CM—cardiomyocyte; EC—endothelial cell; FB—fibroblast; GN—granulocyte; MP—macrophage; T—T cell; W—week.

To specify the major cell types in hearts undergoing cardiac remodeling, we performed clustering and identified 6 major clusters, including cardiomyocytes (CMs) and only a small proportion of MAM-related proteins expressed in endothelial cells (ECs), fibroblasts (FBs), macrophages (MPs), T cells, and granulocytes (GNs), based on their specific molecular markers (Fig. 1A, B). To reveal the role of MAMs in the progression of cardiac hypertrophy, we systemically evaluated the expression of MAM-related proteins in different stages of cardiac hypertrophy. Five stages of cardiac hypertrophy were selected, including 0, 2, 5, 8, and 11 weeks after TAC-induced cardiac hypertrophy, of which 0 weeks indicated normal, 2 weeks indicated hypertrophy with normal ejection fraction (EF), 5 weeks indicated hypertrophy with reduced EF, and 8 and 11 weeks indicated heart failure. The MAM score was derived from the expression levels of some critical MAM-related proteins (Mfn2, Bcap3, Pacs2, Tespa1, Hspa9, Itpr3, SigmaR1, VAPB, Mfn1, Fis1, and Mcu). The MAM score was increased 2 weeks after TAC and gradually reduced as time went on, probably owning to a compensatory effect of MAMs in cardiac hypertrophy (Fig. 1C). The expression of MAMs-related proteins in hearts of mice induced by TAC-induced cardiac hypertrophy was further explored by RT-PCR (Figure S1). The levels of Fis1, Hspa9, Mfn1 and Mfn2 were increased at 2 weeks after TAC, and gradually downregulated in the following weeks after TAC (Figure S1). Further analysis revealed that MAM-related proteins were primarily expressed in CMs, and only a small proportion of MAM-related proteins were expressed in ECs, FBs, MPs, T cells, and GNs (Fig. 1D).

**Figure 2:**
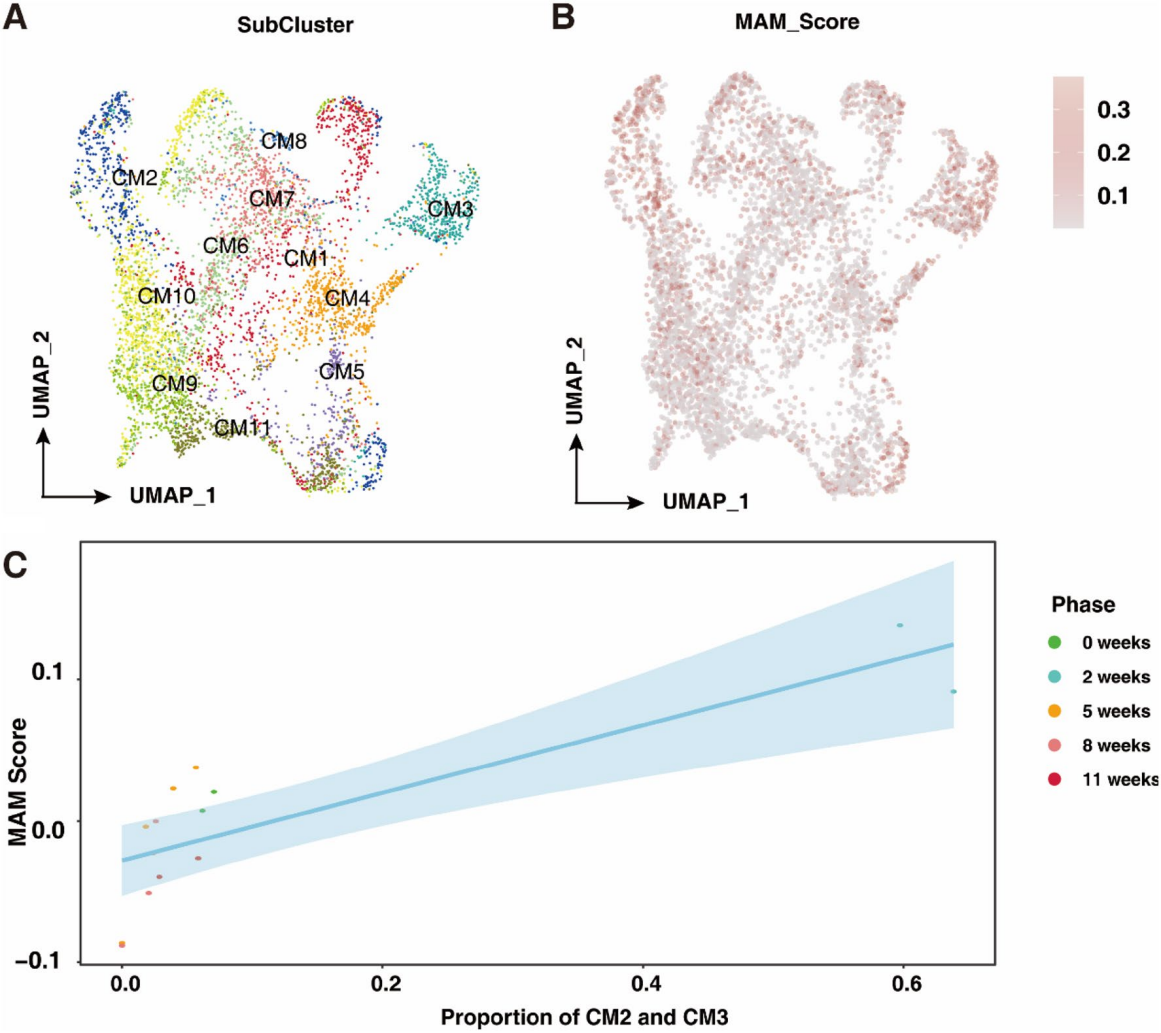
The MAM score was positively related to the proportion of CM2 and CM3 in different stages of cardiac hypertrophy. (**A**) UMAP projection showing CM subtypes in hearts at different stages of cardiac hypertrophy. (**B**) UMAP projection showing MAM scores in each subtype of CMs in hearts at different stages of cardiac hypertrophy. (**C**) Changes in MAM score along percentages of CM2 and CM3.

To precisely locate MAM proteins in cardiomyocytes, we further categorized all cardiomyocytes into 11 subtypes (CM1-11) and observed MAM proteins mainly expressed in the CM2 and CM3 subtypes (Fig. 2A and B). The MAM score was positively related to the proportion of CM2 and CM3 in different stages of cardiac hypertrophy, suggesting the potential role of CM2 and CM3 in the progression of cardiac hypertrophy (Fig. 2C).

### CM2 and CM3 subtypes switching to CM9 and CM10 highly correlates with hypertrophic responses of cardiomyocytes

**Figure 3:**
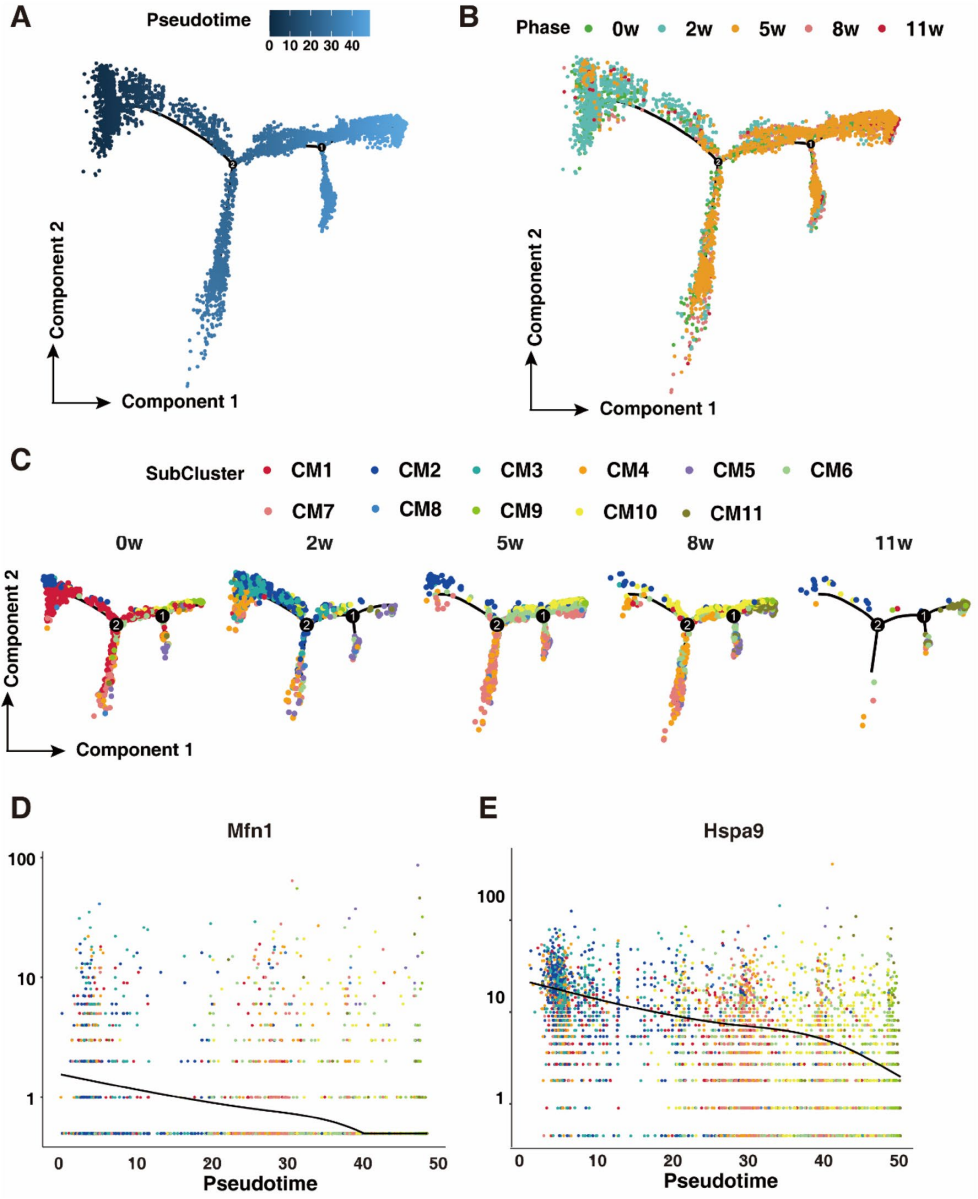
CM2 and CM3 subtype switching to CM9 and CM10 is highly correlated with the hypertrophic responses of cardiomyocytes. (**A**) Pseudotime trajectory analysis of CMs representing the early to terminal transition. (**B**) Pseudotime trajectory analysis of CMs from hearts at different stages of cardiac hypertrophy. (**C**) Pseudotime trajectory analysis of CM subtypes from hearts at different stages of cardiac hypertrophy. (**D**, **E**) Pseudotime expression of Maf1 (**D**) and Hspa9 (**E**), indicating their consecutive downregulation during the process of cardiac hypertrophy.

To further delineate the role of CM2 and CM3 in the progression of cardiac hypertrophy, we aligned cardiomyocytes in each stage with pseudotime trajectory analysis (Fig. 3A, B). Following the initiation of hypertrophy, the subtypes of cardiomyocytes, mainly CM2 and CM3, gradually progressed to CM9, CM10 and CM11, depicting a state with reduced cardiac function (Fig. 3C). Pseudotime alignment of cardiomyocytes also revealed significant downregulation of Mfn1 and Hspa9 levels in each subtype of cardiomyocytes with pseudotime (Fig. 3D, E).

**Figure 4:**
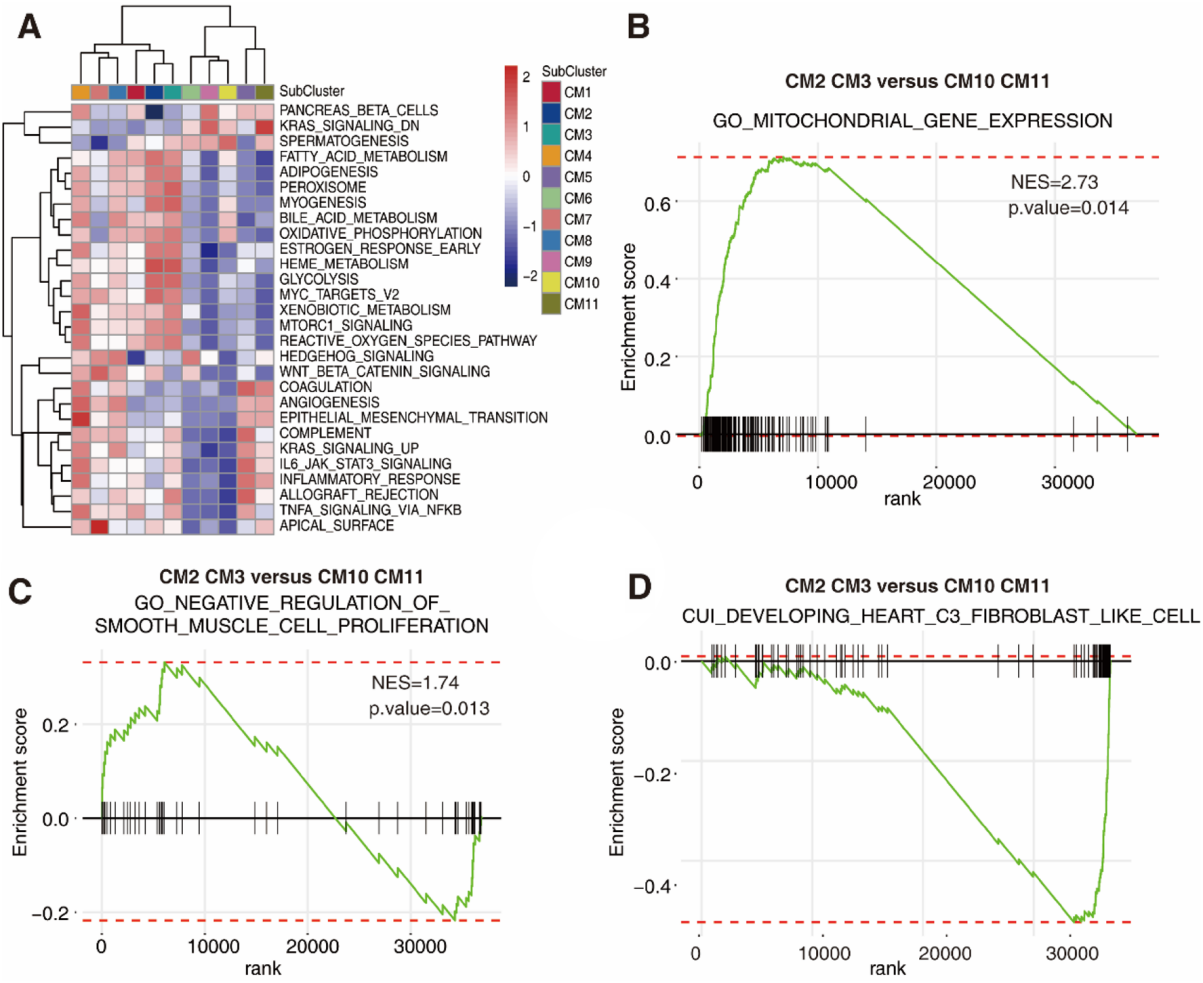
GSVA and GSEA showed the biological processes enriched in each CM subtype. (**A**) GSVA presented the enriched biological processes and signaling pathways in each subtype. (**B**, **C**, **D**) GSEA of CM2, CM3, CM10, and CM11 revealed significant enrichment in mitochondrial gene expression (**B**), negative regulation of smooth muscle cell proliferation (**C**), and fibroblast-like cell signatures (**D**) in each subtype.

Gene set variation analysis (GSVA) revealed that the CM1 subtype was predicted to be involved in fatty acid metabolism and oxidative phosphorylation (Fig. 4A). The CM2 and CM3 populations were concurrently characterized by glycolysis, myogenesis, cellular respiration and ATP metabolic processes, while CM10 and CM11 were negatively enriched in these biological processes (Fig. 4A). CM4 was characterized by epithelial-mesenchymal transition and angiogenesis. CM5, by contrast, displayed an inflammatory response and IL6-JAK-STAT3 signaling (Fig. 4A). Therefore, the transition of CMs from the CM2 and CM3 subtypes to the CM10 and CM11 subtypes suggests a decline in oxidative phosphorylation and energy supply. Gene set enrichment analysis (GSEA) showed that genes upregulated in the CM2 and CM3 subtypes after TAC were significantly enriched in mitochondrial gene expression (Fig. 4B). Meanwhile, the CM2 and CM3 subtypes are characterized by negative regulation of smooth muscle cell proliferation compared with the CM10 and CM11 subtypes (Fig. 4C). The shift of CM2 and CM3 to CM10 and CM11 displayed a fibroblast-like phenotype, indicating that the myocardium underwent fibroblast-like changes in the progression of cardiac hypertrophy (Fig. 4D).

### Transcription factors that mediate the phenotypical transition of cardiomyocytes in cardiac hypertrophy

**Figure 5:**
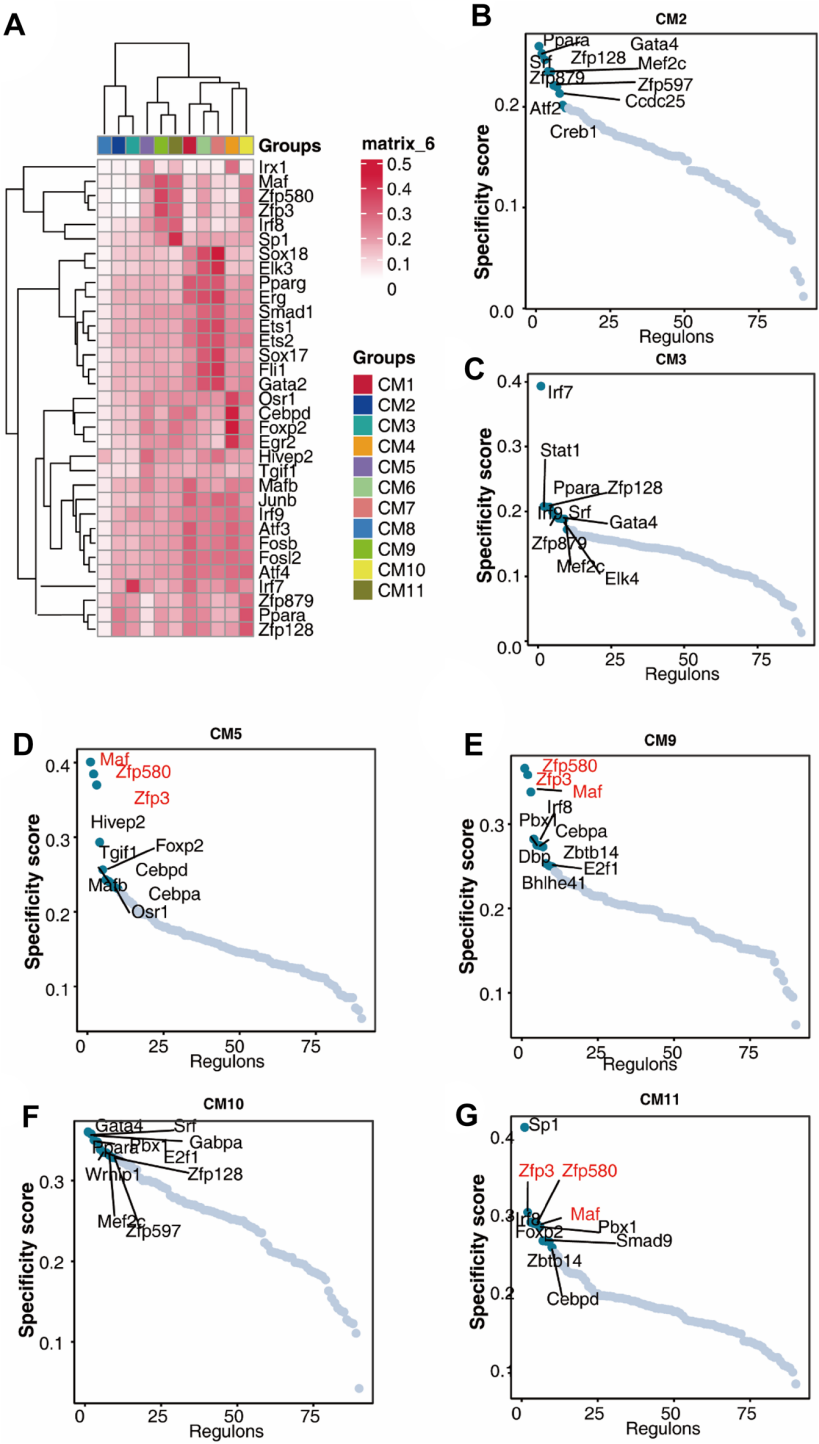
Transcription factors that mediate the phenotypical transition of cardiomyocytes in cardiac hypertrophy. (**A**) Transcription factors enriched in each subtype. (**B**, **C**, **D**, **E**, **F**, **G**), Specificity score of transcription factors in CM2 (**B**), CM3 (**C**), CM5 (**D**), CM9 (**E**), CM10 (**F**), and CM11 (**G**).

To reveal the transcription factors that mediate the phenotypical transition of cardiomyocytes during cardiac hypertrophy, we analyzed the expression of specific transcription factors in each CM subtype. Gata4, Mef2c and Tbx5 are the main modulators occupying the priority of the transcriptional hierarchy of the cardiac gene regulatory networks. Regarding cardiac development, precise spatial and temporal control of these three factors is a prerequisite for delicate execution of cardiac developmental processes. PPARα is considered to be important in cardiac hypertrophy and fibrosis. As shown in Fig. 5A, CM2 and CM3 shared a similar pattern of transcription factor expression, such as Gata4, PPAR α, Mef2c, and Zfp879, implying normal cardiac function in these subtypes (Fig. 5A, B, C). Zfp580 is probably related to ventricular remodeling after myocardial I/R injury by involving TGF-β1-induced cardiomyocyte hypertrophy. Consistently, we observed that Zfp580 and Zfp3 were specifically upregulated in the CM5, CM9 and CM11 subtypes, implicating a functional switch in different disease stages (Fig. 5D, E, F, G). Taken together, the difference in transcription factors in each subtype may serve as the driving force that promotes subtype transformation.

### MAMs that correlated with cardiac hypertrophy revealed by scWGCNA

**Figure 6:**
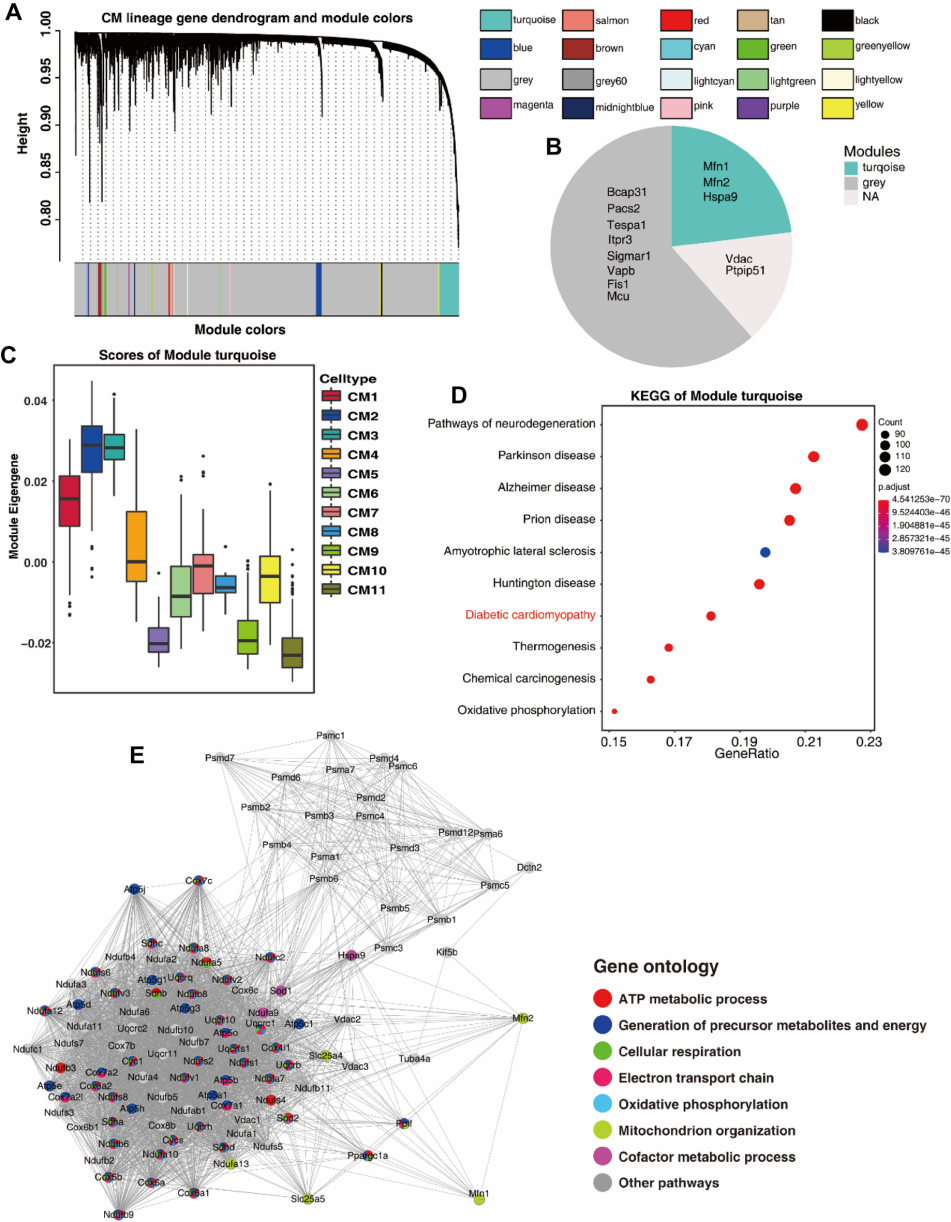
Transcription factors that mediate the phenotypical transition of cardiomyocytes in cardiac hypertrophy. (**A**) The cluster dendrogram of genes in GSE120064. Branches of the clustered dendrogram of the most connected genes. (**B**) MAM-related genes were mainly clustered into turquoise and gray modules. (**C**) Score of module turquoise in each cardiomyocyte subtype. (**D**, **E**) KEGG and GO analysis of the turquoise module^20,35^. “NS” indicates that these genes were not detected in single cells.

The scWGCNA package was used to screen module genes associated with cardiac hypertrophy. Different module genes were clustered with a topological similarity strategy. Eventually, twenty modules developed after clustering, and we noticed that the turquoise module was closely correlated with cardiac hypertrophy (Fig. 6A). We found that Mfn1, Mfn2, and Hspa9 were present in the turquoise module. Other MAMs, such as Bcap31, Pacs2, Tespa1, and Itpr3, were mainly present in the gray module (Fig. 6B). The turquoise module was highly expressed in the CM1, CM2 and CM3 subtypes and was reduced in the CM10 and CM11 subtypes (Fig. 6C). To explore the potential functions of the genes within the turquoise module, we performed GO and KEGG pathway analyses and observed the most significant KEGG pathways and GO terms in Fig. 6D and E^19–21^. Genes in the turquoise module were mainly enriched in diabetic cardiomyopathy, neurodegeneration, Parkinson’s disease, and so on. Moreover, genes in the turquoise module were preferentially enriched in ATP metabolic process, generation of precursor metabolites, cellular respiration, electron transport chain and oxidative phosphorylation (Fig. 6E).

## Discussion

The pathogenesis of cardiac hypertrophy is sophisticated, and various signaling mechanisms and cellular processes are implicated in the pathogenesis of cardiac hypertrophy^22^. Escalating studies have revealed the function of MAMs in the regulation of cardiovascular diseases and could potentially serve as therapeutic targets in cardiovascular diseases^23,24^. In this study, we observed that the MAM score was increased 2 weeks after TAC and gradually reduced 5, 8, and 11 weeks after TAC. MAM-related proteins were preferentially enriched in cardiomyocytes, more precisely, in the CM2 and CM3 subtypes. The CM2 and CM3 subtypes switched to the CM9 and CM10 subtypes, which highly correlate with the hypertrophic responses of cardiomyocytes. Transcription factors enriched in these subtypes were responsible for the functional transformation of these subtypes.

MAMs are closely involved in cardiac diseases because of their critical functions in lipid metabolism, calcium transfer, ROS, ER stress, mitochondrial dynamics and mitophagy^1^. The MAM protein Mfn2 is downregulated in cardiac hypertrophy induced by pressure overload^25^. Mfn1/2 inhibition was detected in guinea pig HF models. The modulation of MAM-related proteins could affect the progression of cardiac hypertrophy^26^. For instance, cardiac-specific deletion of Mfn2 promoted cardiac hypertrophy accompanied by moderate diastolic dysfunction as well as alterations in smooth endoplasmic reticulum-mitochondrial contact^27^. Activation of Sig-1R could protect against abdominal aortic banding or TAC-induced cardiac hypertrophy^28^. Therefore, MAMs are implicated in the pathogenesis of cardiac hypertrophy, and their precise role in cardiac hypertrophy remains elusive. In this study, we conducted a comprehensive analysis of the process of cardiac hypertrophy, including mild, moderate hypertrophy, and aggravated heart failure, to further illustrate the alterations in MAM-related proteins during these phases. Interestingly, the MAM score initially increased at 2 weeks after TAC and then gradually decreased in the following several weeks, indicating the potential compensatory effect of MAMs in the process of cardiac hypertrophy. MAMs were predominantly expressed in the CM2 and CM3 subtypes and correlated with the expression of MAM proteins.

Further exploration of the CM2 and CM3 subtypes revealed that these two subtypes underwent phenotypic switching in the process of cardiac hypertrophy. Along with sustained pressure overload, the CM2 and CM3 subtypes switched to the CM9 and CM10 subtypes. Meanwhile, the expression of Mfn1 and Hspa9 gradually decreased during pseudotime analysis. Mfn1 is reported to alleviate cardiomyocyte apoptosis and myocardial infarction size in mice with ischemia/reperfusion injury^29^. Downregulation of Mfn1 has been observed in cardiac hypertrophy animal models, depicting its protective effect in cardiac hypertrophy. Hspa9, a chaperone also located on MAM, directly modulates calcium transfer from the ER to the mitochondria, which plays a critical role in cardiac excitation–contraction coupling and calcium overload during heart failure^30^. Therefore, abnormal regulation of Mfn1 and Hspa9 might have detrimental effects on the normal function of the heart and potentially induce cardiac hypertrophy. Genes highly enriched in CM2 and CM3 were mitochondrial-related genes, including glycolysis, myogenesis, cellular respiration and ATP metabolic process. These oxidative phosphorylation processes were downregulated in the CM10 and CM11 subtypes. The fibroblast-like process and inflammatory response took the place of the original metabolic processes, indicating that the myocardium underwent fibroblast-like changes in the progression of cardiac hypertrophy. As previously acknowledged, fibroblasts play a critical role in inducing cardiomyocyte hypertrophy in vivo through paracrine secretion of growth factors and other signaling molecules, which is quite relevant to our analysis^31^.

Further investigations on the driving force that promotes the transition of CM2 and CM3 into CM10 and CM11 were performed to display the involved transcription factors enriched in each subtype. CM2 and CM3 shared similar expression patterns of transcription factors, such as Gata4, PPARα, Mef2c, and Zfp879, which were negatively enriched in the CM10 and CM11 subtypes. Gata4 and Mef2c are the main modulators occupying the top of the transcriptional hierarchy of the cardiac gene regulatory networks^32^. Mef2 was previously identified as a fundamental transcriptional regulator in both cardiogenesis and cardiac hypertrophy and is significantly activated in hypertensive hearts. Mef2c silencing alleviates pressure-induced left ventricular hypertrophy by modulating the mTOR/S6K pathway. In the precise control of cardiac development, precise spatial and temporal control of these three factors is a prerequisite for delicate execution of cardiac developmental processes. PPARα plays an important role in cardiac metabolism and anti-inflammation, which is considered to be important in cardiac hypertrophy and fibrosis^33^. Moreover, the cardiogenic transcription factors Mef2c, Gata4, and Tbx5 can directly reprogram fibroblasts to induce cardiac-like myocytes. Therefore, the enrichment of these transcription factors sustain normal cardiac function. Deficiency of these transcription factors might affect the downstream genes of these factors, which potentially contribute to hypertrophic responses. Zfp580 and Zfp3 were specifically upregulated in the CM5, CM9 and CM11 subtypes and may potentially act as modulators of subtype transformation. We screened and identified two key proteins, Zfp580 and Zfp3, that play an important regulatory role in myocardial hypertrophy. Due to space constraints in this study, we did not include functional experiments of these two proteins. Zfp580 is a novel Cys2-His2 zinc-finger transcription factor that has an anti-apoptotic role in myocardial cells. Zfp580 is probably associated with ventricular remodeling after myocardial I/R injury by involving TGF-β1-induced cardiomyocyte hypertrophy^34^. for each annotated gene associated with heart failure trait. ZNF146 and Zfp3 are potential genetic contributors for incident heart failure with reduced ejection fraction. Therefore, we presume that the enrichment of Zfp580 is likely associated with ventricle remodeling during cardiac hypertrophy.

Taken together, MAM-related proteins preferentially accumulated in cardiomyocytes during the initial stage of cardiac hypertrophy and underwent a gradual decline as the disease progressed. The proportion of initial cardiomyocyte subtypes (CM2 and CM3) was positively correlated with the MAM score. These subtypes went through a functional switch during cardiac hypertrophy. In addition, transcription factors in each subtype changed significantly across the process of cardiac hypertrophy. Overall, we identified cardiomyocyte subtype transformation and the potentially involved critical transcription factors, which may serve as therapeutic targets in combating cardiac hypertrophy.

## Supplementary Information

Supplementary Information.

## Data Availability

The datasets analyzed during the current study are available in the Gene Expression Omnibus (GEO) database (GSE120064).

## References

[1] Mao L (2022). Heart-targeting exosomes from human cardiosphere-derived cells improve the therapeutic effect on cardiac hypertrophy. J. Nanobiotechnol..

[2] Woodcock EA, Matkovich SJ (2005). Cardiomyocytes structure, function and associated pathologies. Int. J. Biochem. Cell Biol..

[3] Chen S (2022). Melatonin activates the Mst1-Nrf2 signaling to alleviate cardiac hypertrophy in pulmonary arterial hypertension. Eur. J. Pharmacol..

[4] Ji S (2022). NO_2_ exposure contributes to cardiac hypertrophy in male mice through apoptosis signaling pathways. Chemosphere.

[5] Yang LL (2022). E3 ubiquitin ligase RNF5 attenuates pathological cardiac hypertrophy through STING. Cell Death Dis..

[6] Wang K (2022). IRX2 activated by jumonji domain-containing protein 2A is crucial for cardiac hypertrophy and dysfunction in response to the hypertrophic stimuli. Int. J. Cardiol..

[7] Facundo H (2017). Mitochondria and cardiac hypertrophy. Adv. Exp. Med. Biol..

[8] Rosca MG, Tandler B, Hoppel CL (2013). Mitochondria in cardiac hypertrophy and heart failure. J. Mol. Cell Cardiol..

[9] Liang X (2015). Characterization of endonuclease G and mitochondria-sarcoplasmic reticulum-related proteins during cardiac hypertrophy. Pharmazie.

[10] Liu J, Yang J (2022). Mitochondria-associated membranes: A hub for neurodegenerative diseases. Biomed. Pharmacother..

[11] Gao P, Yan Z, Zhu Z (2020). Mitochondria-associated endoplasmic reticulum membranes in cardiovascular diseases. Front. Cell Dev. Biol..

[12] Luan Y (2021). Structure and function of mitochondria-associated endoplasmic reticulum membranes (MAMs) and their role in cardiovascular diseases. Oxid. Med. Cell Longev..

[13] Nieblas B, Perez-Trevino P, Garcia N (2022). Role of mitochondria-associated endoplasmic reticulum membranes in insulin sensitivity, energy metabolism, and contraction of skeletal muscle. Front. Mol. Biosci..

[14] Wu S, Zou MH (2019). Mitochondria-associated endoplasmic reticulum membranes in the heart. Arch. Biochem. Biophys..

[15] Wang Y (2022). Transient receptor potential vanilloid type 1 protects against pressure overload-induced cardiac hypertrophy by promoting mitochondria-associated endoplasmic reticulum membranes. J. Cardiovasc. Pharmacol..

[16] Houser SR (2014). Role of RyR_2_ phosphorylation in heart failure and arrhythmias: protein kinase A-mediated hyperphosphorylation of the ryanodine receptor at serine 2808 does not alter cardiac contractility or cause heart failure and arrhythmias. Circ. Res..

[17] Rieusset J (2018). The role of endoplasmic reticulum-mitochondria contact sites in the control of glucose homeostasis: an update. Cell Death Dis..

[18] Hanzelmann S, Castelo R, Guinney J (2013). GSVA: Gene set variation analysis for microarray and RNA-seq data. BMC Bioinform..

[19] Kanehisa M, Goto S (2000). KEGG: Kyoto encyclopedia of genes and genomes. Nucleic Acids Res..

[20] Kanehisa M (2019). Toward understanding the origin and evolution of cellular organisms. Protein Sci..

[21] Kanehisa M (2023). KEGG for taxonomy-based analysis of pathways and genomes. Nucleic Acids Res..

[22] Graham D (2009). Mitochondria-targeted antioxidant MitoQ10 improves endothelial function and attenuates cardiac hypertrophy. Hypertension.

[23] Stone SJ (2009). The endoplasmic reticulum enzyme DGAT_2_ is found in mitochondria-associated membranes and has a mitochondrial targeting signal that promotes its association with mitochondria. J. Biol. Chem..

[24] Wu S (2017). Binding of FUN14 domain containing 1 with inositol 1,4,5-trisphosphate receptor in mitochondria-associated endoplasmic reticulum membranes maintains mitochondrial dynamics and function in hearts in vivo. Circulation.

[25] Fang L (2007). Down-regulation of mitofusin-2 expression in cardiac hypertrophy in vitro and in vivo. Life Sci..

[26] Yu H (2011). Mitofusin 2 inhibits angiotensin II-induced myocardial hypertrophy. J. Cardiovasc. Pharmacol. Ther..

[27] Han S (2021). The role of Mfn_2_ in the structure and function of endoplasmic reticulum-mitochondrial tethering in vivo. J. Cell Sci..

[28] Bhuiyan MS, Fukunaga K (2009). Stimulation of sigma-1 receptor signaling by dehydroepiandrosterone ameliorates pressure overload-induced hypertrophy and dysfunctions in ovariectomized rats. Expert Opin. Ther. Targets.

[29] Hall AR (2016). Hearts deficient in both Mfn1 and Mfn2 are protected against acute myocardial infarction. Cell Death Dis..

[30] Yuan M (2022). IP3R1/GRP75/VDAC1 complex mediates endoplasmic reticulum stress-mitochondrial oxidative stress in diabetic atrial remodeling. Redox Biol..

[31] Kamo T, Akazawa H, Komuro I (2015). Cardiac nonmyocytes in the hub of cardiac hypertrophy. Circ. Res..

[32] Wang L (2015). Stoichiometry of Gata4, Mef2c, and Tbx5 influences the efficiency and quality of induced cardiac myocyte reprogramming. Circ. Res..

[33] Planavila A (2011). Sirt1 acts in association with PPARalpha to protect the heart from hypertrophy, metabolic dysregulation, and inflammation. Cardiovasc. Res..

[34] Miao, J., et al., *Effects of ZFP580 on Ventricular Remodeling after Myocardial Ischemia/reperfusion in Rats*. **33**(3), 262–266 (2017). 10.12047/j.cjap.5544.2017.064 29931944

[35] Ogata H (1999). KEGG: Kyoto encyclopedia of genes and genomes. Nucleic Acids Res..

