# Supplementary material for: Single-cell transcriptional profiling of hearts during cardiac hypertrophy reveals the role of MAMs in cardiomyocyte subtype switching
Source: Sci Rep. 2023 May 23;13:8339. doi: 10.1038/s41598-023-35464-2 (PMC10205799; doi:10.1038/s41598-023-35464-2)
Supplement: Supplementary file 1 — Supplementary Information. [file 41598_2023_35464_MOESM1_ESM.pdf]

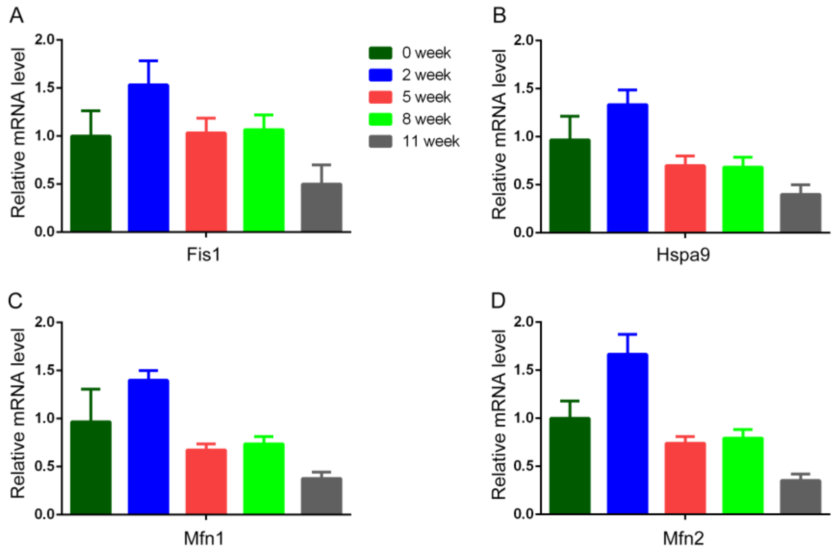

Figure S1. The relative expression level of MAM-related proteins. The level of Fis1 (A), Hspa9 (B), Mfn1 (C), Mfn2 (D) in the hearts of mice induced by TAC.
